# Protective Effects of Amlodipine Pretreatment on Contrast-Induced Acute Kidney Injury And Overall Survival In Hypertensive Patients

**DOI:** 10.3389/fphar.2020.00044

**Published:** 2020-02-11

**Authors:** Wen-jun Yin, Ling-yun Zhou, Dai-yang Li, Yue-liang Xie, Jiang-lin Wang, Shan-ru Zuo, Kun Liu, Can Hu, Ge Zhou, Lin-hua Chen, Hui-qing Yang, Xiao-cong Zuo

**Affiliations:** ^1^ Department of Pharmacy, The Third Xiangya Hospital of Central South University, Changsha, China; ^2^ Center of Clinical Pharmacology, The Third Xiangya Hospital of Central South University, Changsha, China

**Keywords:** contrast media, contrast-induced acute kidney injury, acute kidney injury, risk factor, amlodipine, hypertension

## Abstract

**Backgroud:**

Contrast-induced acute kidney injury (CI-AKI) is the most common adverse reaction caused by contrast media, which has been reported to prolong hospitalization and increase mortality and morbidity. The hypertensive population has proved susceptible to CI-AKI. Unfortunately, no therapeutic has been shown to prevent and cure CI-AKI effectively. A few studies have shown the protection of amlodipine on renal function, but the relationship between amlodipine and CI-AKI in hypertensive group is unknown, we aimed to study the effects of amlodipine on CI-AKI and overall survival in a large Chinese hypertensive cohort.

**Methods:**

A retrospective, matched, cohort study was conducted among adults hospitalized at the Third Xiangya Hospital of Central South University from October 2007 to May 2017. CI-AKI was the primary end point of the trial, time-related all-cause mortality (including in-hospital) and length of hospital stay were the secondary end points. Propensity Score Matching was used to reduce the effect of selection bias and potential confounding.

**Results:**

868 patients with and 1,798 ones without amlodipine before contrast administration were included. The incidence of CI-AKI was 10.50%. The unadjusted, adjusted, and propensity‐score matched incidence of CI-AKI were lower in patients treated with amlodipine (OR, 0.650; *P *= 0 .003; OR, 0.577; *P *= 0.007; OR, 0.687; *P *= 0.015, respectively), and the same results were found in the subgroups of diabetes, chronic kidney disease (CKD), non-CKD, low-osmolar, and elderly. Moreover, amlodipine reduced hospital stay, whether matched or not (7.08 ± 7.28 vs 7.77 ± 7.82, *P *= 0.027, before matching; vs 7.81 ± 7.58, *P *= 0.040, after matching). 1,046 patients finished follow-up including 343 amlodipine users and 703 non-users. The overall mortality was significantly lower among amlodipine users (10.79%) than controls (16.07%), the significant difference was found in survival between them (*P *= 0.024, log-rank test), amlodipine was associated with longer overall survival [HR, 0.623; 95% CI (0.430–0.908), *P *= 0.014].

**Conclusion:**

In conclusion, we first found amlodipine treatment before contrast exposure played a role in protecting hypertensive patients from CI-AKI and prolonging survival.

## Introduction

Contrast-induced acute kidney injury (CI-AKI) is the most common clinical complication of the intravascular administration of contrast media (CM) which can prolong hospitalization and increase morbidity and mortality. (Tepel et al., 2006) What's more, CI-AKI has become the third prevalent cause of all hospital-acquired renal failure, accounting for 10%. (Nash et al., 2002) According to the Contrast Media Safety Committee, CI-AKI was defined as an increase in serum creatinine (Scr) of 0.5 mg/dl (44.2 μmol/L) or 25% relative increase in Scr from the baseline value to 72 hours after exposure to CM.

Unfortunately, there is no strategy to effectively prevent and cure CI-AKI. Therefore, we should attach significance to identifying high-risk CI-AKI groups for earlier preventive measures as soon as possible to reduce CI-AKI. Hypertension is also a major risk factor of CI-AKI and increases the risk of two times, (Bartholomew et al., 2004) and it is a leading cause of chronic kidney disease (CKD), kidney disease progression, and end-stage kidney disease. (Klag et al., 1996; Fox et al., 2004; Carey et al., 2018) According to the 2015 Report on the Status of Nutrition and Chronic Diseases in China, the incidence of hypertension is greatly high, approximately 25.2% of adults suffer from hypertension. (Zhang et al., 2017) Although the hypertensive population is a huge group with high-risk of CI-AKI, CI-AKI in hypertensive patients has not been studied.

The precise mechanisms underlying CI-AKI are not well understood. However, the toxicity effect of CM on the tubular epithelial cells due to apoptosis has been reported to be related to the pathogenesis of CI-AKI. And the apoptosis of renal vasoconstriction and tubular induced by CM have been considered relevant to changes in calcium physiology. (Neumayer et al., 1989; Russo et al., 1990; Yang et al., 2008; Yang et al., 2013) Intracellular calcium overload followed by reactive oxygen species overproduction and caspase-3 overexpression was found to play a key role in renal tubular cytotoxicity induced by CM. (Duan et al., 2000) Although the physiological and pathophysiological mechanisms of Ca^2+^ overload in ischemic kidney and CI-AKI have not been fully elucidated, there is evidence that the increase of Ca^2+^ in cytoplasm may be an important mediator of epithelial cell apoptosis and necrosis. (Wilson et al., 1984) Therefore, in theory, calcium channel blockers (CCB) can selectively block Ca^2+^ channels in cell membranes, prevent Ca^2+^ from entering cells, and have a protective effect on CI-AKI.

Amlodipine, one of the most common used CCB drugs, is a dihydropyridine CCB that inhibits the slow channel transmembrane influx of calcium ions into vascular smooth muscle and into cardiac muscle with fewer side effects and a longer half-life than most antihypertensives, enabling once-daily dosing (Haria and Wagstaff, 1995). Some studies have shown that amlodipine has a protective effect on the kidney. In basic experimental study, amlodipine can inhibit the HKC apoptosis and protect the renal tubule cell from injury induced by meglumine diatrizoate, in the amlodipine group, the cell viability increased significantly, LDH levels decreased, and the apoptosis was lower than that of the model group, Bax protein expression and caspase 3 activity decreased. (Zhou et al., 2007) Duan et al. found calcium load played a role in producing renal function impairment induced by diatrizoate meglumine, and amlodipine protected the renal tissue from nephrotoxicity induced by diatrizoate. (Duan et al., 2000) Combined aliskiren and amlodipine reduced albuminuria via reduction in renal inflammation in diabetic rats. In clinical research, amlodipine offers protection against CI-AKI in elderly patients with coronary heart disease, (Owen et al., 2014) and the combination therapy with amlodipine and atorvastatin may exert additional beneficial effects on renal and vascular damages as well as blood pressure (BP) profile in addition to BP lowering in hypertension with CKD. (Azushima et al., 2014) However, it is unclear whether amlodipine can protect CI-AKI in hypertensive population and the long-term outcome of amlodipine in hypertensive population with contrast administration.

The purpose of this study was to determine the effect of amlodipine before contrast exposure on the CI-AKI and overall survival in a large Chinese hypertensive cohort.

## Materials and Methods

### Ethics Statement

The Medical Ethical Committee in the Third Xiangya Hospital of Central South University approved this study (No. 2016-S160). All subjects were anonymized, thus the provision of informed consent was not required. This study complied with the ethical guidelines of the 1975 Declaration of Helsinki and Strengthening the Reporting of Observational Studies in Epidemiology guidelines.

### Patient Population

A retrospective, matched, cohort study was conducted among adults hospitalized at the Third Xiangya Hospital of Central South University from October 2007 to May 2017. Patients were included if they were administered with CM procedure including coronary angiography, percutaneous coronary intervention, received intravenous CM such as for enhanced CT and intravascular surgery, and their admitting diagnosis is recognized as “hypertension”, identified by the electronic medical record system. Patients with the following conditions were excluded: 1) preprocedure estimated glomerular filtration rate (eGFR) under 15 ml/(min 1.73 m^2^); 2) treated with CCB drugs other than amlodipine or levamlodipine; 3) the dosage regimen is not 2.5 mg/qd for levamlodipine or not 5.0 mg/qd for amlodipine; 4) age ≤ 18. Their detailed demographic and clinical characteristics were obtained from the structured hospital information system of The Third Xiangya Hospital (Changsha, China) included demographics, baseline and postoperative Scr, preoperative hemoglobin, diastolic blood pressure, systolic blood pressure, complication, discharge diagnosis, Killip scores, and medications included diuretic, ACEI, ARB, βblockers, aspirin, and alprostadil. In the survival analysis, we excluded the patients with missing or replacing telephone numbers and who refused to be followed up.

### Study End Points and Definitions

The primary end point of the trial was the incidence of CI-AKI, the secondary end point was time-related all-cause mortality (including in- hospital) and length of hospital stay. CI-AKI was defined as an increase in Scr of 0.5 mg/dl (44.2 μmol/L) or 25% relative increase in Scr from the baseline value to 72 hours after exposure to CM in the absence of alternative causes for acute kidney injury. The earliest Scr concentration within 14 days prior to contrast was defined as baseline, and the highest Scr concentration within 72 hours after contrast was used as follow-up Scr to evaluate the incidence of CI-AKI. CKD was defined as eGFR < 60 ml/(min 1.73 m^2^) calculated by the Modification of Diet in Renal Disease equation. (Matsushita et al., 2012; Palevsky et al., 2013) Anemia was defined as hemoglobin concentration <13 g/dl for men and <12 g/dl for women. (Nutritional Anaemias, 1968) The aged were equal to those over 60 years old.

### Statistical Analysis

All of the analyses were performed using IBM SPSS Version 22.0 (SPSS, Inc., Chicago, IL) and R (version3.5.1). Continuous variables were presented as means and standard deviations (SDs). A t-test was used to compare the normally distributed continuous variables; otherwise, the Mann-Whiney U-test was performed. Categorical variables were performed by chi-squared test. Logistic regression analysis was performed to evaluate the efficacy of amlodipine treatment on CI-AKI, adjusting for various potential prognostic and confounding factors. A two-tailed value of *P* < 0.05 was established as the threshold of statistical significance.

In order to reduce the impact of selection bias and potential confounding in this study, we rigorously adjusted the differences in renal function and diabetes mellitus, which has been reported as independent risk factors for CI-AKI, by propensity score analysis between the two groups (amlodipine and no amlodipine) to assess the outcomes of CI-AKI. Propensity scores were calculated using logistic regression of age, sex, CKD, diabetes, baseline Scr, baseline GFR, since renal insufficiency and diabetes were reported as independent risk factors of CI-AKI in the previous studies. Propensity Score Matching is a technique that attempts to approximate a random experiment, eliminating many of the problems and reducing the bias due to confounding variables that come with observational data analysis by matching treated patients to controls that were similarly likely in the same group. The possibility of bias occurs because some characteristics instead of the effect of the treatment decides the apparent difference in outcome between these two groups that received the treatment versus those that did not. The randomization enables agonic estimation of curative effects in randomized experiments; according to the law of large numbers, randomization means that treatment-groups will be balanced on average on each covariate. While in observational studies, the treatments to research subjects are usually assigned at nonrandom. In order to imitate randomization, a unit sample which received the treatment that is equivalent on all observed covariates to a unit sample that did not receive the treatment is created by matching. (Ho et al., 2007) In this study, propensity matching was performed with a 1:1 genetic matching for case and control subjects in which the nearest neighbor was selected. (Diamond and Sekhon, 2013) The comparative risk of outcome was further adjusted for a conditional logistic-regression model, the adjusted variables included age, sex, body mass index (BMI), baseline eGFR, Scr, CKD, diabetes, Killip III, systolic blood pressure (SBP), diastolic blood pressure (DBP), hyperlipidemia, anemia, aspirin, diuretic, angiotensin-converting enzyme inhibitors (ACEI), angiotensin receptor blockers (ARB), β blockers, and alprostadil. To further study the reliability of the results, we also carried out subgroup analysis in CKD, diabetes, and the aged population. In addition, the effects of amlodipine dosage and duration were studied.

All patients were followed up until occurrence of death, end of the study period, or loss to follow-up. Time to all-cause mortality was analyzed using Cox proportional hazards models in our cohorts and hazard ratios with 95% CIs were estimated adjusting for baseline stratification factors. Survival time was calculated as time from contrast administration to death, loss to follow-up, or end of study period. Survival time was censored on December 26, 2018 or at the time a patient was lost to follow-up. The association of amlodipine and death were obtained by using Kaplan–Meier curves over the entire study period. Hazard ratios and odds ratios were reported relative to study participants without amlodipine.

## Results

### Patient Characteristics

**Figure 1:**
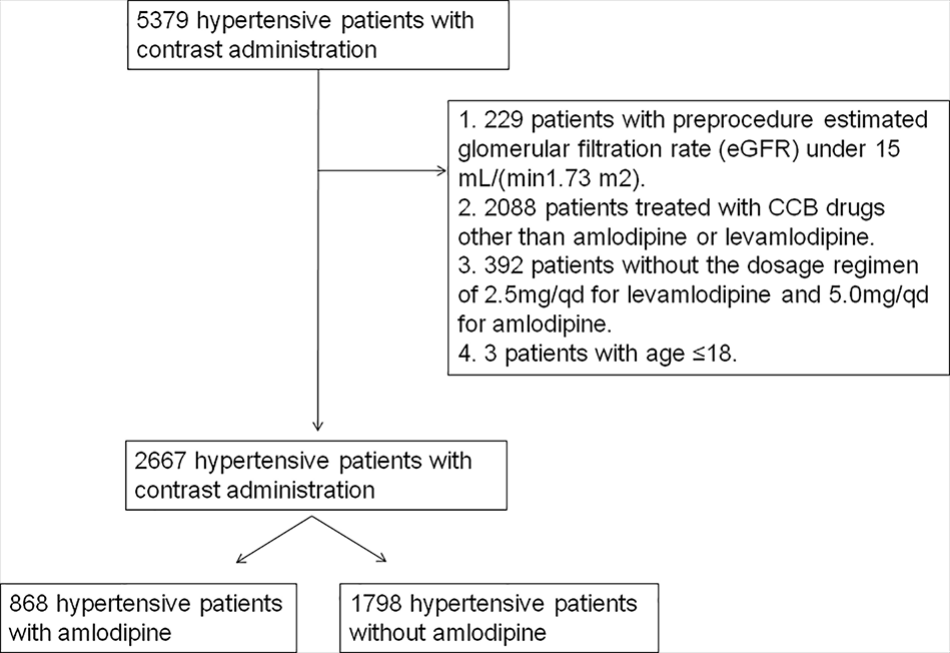
Flow chart depicting number of patients who were included in analysis after exclusion criteria.

Among a total of initial 5,379 hypertensive patients with contrast administration, there were 3 juveniles (<18 years), 2,088 treated with CCB drugs other than amlodipine or levamlodipine, 229 with preprocedure eGFR under 15 ml/(min 1.73 m^2^), and 392 without the dosage regimen of 2.5 mg/qd for levamlodipine or 5.0 mg/qd for amlodipine. After excluding the above-mentioned participants 2,666 patients were enrolled in the final analysis. The mean age of the total population was 63.53±9.45 years, and 1,647 (61.78%) of them were males. Of these, 868 patients received amlodipine (including levamlodipine) and 1,798 controls were selected. With the use of propensity score matching, 868 matched controls were identified. **Figure 1** showed the number of patients included in analysis after applying for exclusion criteria. The baseline characteristics of the study population separated by amlodipine, controls, and matched controls are presented in **Table 1**. The Scr levels were significantly higher prior to matching and the CKD, diabetes, and male were significant prevalent in the amlodipine group in comparison to controls. After matching, these differences between the two groups were reduced. About 70% of patients used low-osmolar, 17% used iso-osmolar in this cohort.

**Table 1:** Baseline characteristics before and after propensity-score matching.

Characteristic	Amlodipine group (n = 868)	Control group (n = 1,798)	Matched control group (n = 868)	*P_1_*	*P_2_*
**Age**					
Mean (years)	63.66±9.51	63.46±9.45	63.79±9.92	0.613	0.778
**Distribution**					
<60 years	203 (23.39%)	696 (38.54%)	242 (27.88%)		
≥60 years	665 (76.61%)	1,102 (61.29%)	626 (72.12%)	<0.001	0.032
**Sex (%)**					
Male	537 (61.87%)	1,110 (61.74%)	542 (62.44%)	0.032	0.850
Female	331 (38.13%)	688 (38.26%)	326 (37.56%)		
**Body mass index, kg/m^2^**	24.62±3.45	24.36±3.28	24.34±3.31	0.062	0.094
**Scr**	94.50±47.75	90.27±47.75	95.50±52.71	0.032	0.693
**GFR**	82.18±38.48	84.25±38.48	80.48±32.08	0.194	0.377
**Contrast type**					
Low-osmolar	673 (77.53%)	1,474 (82.39%)	708 (81.57%)	0.007	0.037
Iso-osmolar	151 (17.40%)	298 (16.66%)	152 (17.51%)	0.595	0.950
High-osmolar	1 (0.12%)	26 (1.45%)	8 (0.92%)	0.001	0.019
**Diabetes**	296 (34.10%)	501 (27.86%)	285 (32.83%)	0.001	0.576
**Anemia**	352 (40.55%)	823(45.77%)	436 (50.23%)	0.011	<0.001
**Hyperlipidemia**	92 (10.60%)	136 (7.56%)	73 (8.41%)	0.010	0.088
**Killip III**	94 (10.83%)	152 (8.45%)	76 (8.76%)	0.109	0.146
**Medications**					
Diuretic	159 (18.32%)	372 (20.69%)	188 (21.66%)	0.196	0.082
ACEI	417 (48.04%)	776 (43.16%)	380 (43.78%)	0.553	0.068
ARB	174 (20.04%)	227 (12.63%)	104 (11.98%)	<0.001	<0.001
β-blockers	473 (54.49%)	848 (47.16%)	414 (47.70%)	<0.001	0.005
Aspirin	552 (63.59%)	957 (53.23%)	465 (53.57%)	0.003	<0.001
Alprostadil	193 (22.24%)	351 (19.52%)	166 (19.12%)	0.087	0.123

eGFR, estimated glomerular filtration rate; SBP, systolic blood pressure; DBP, diastolic blood pressure; CKD, chronic kidney disease [defined as eGFR <60 mL/(min 1.73 m^2^)]; ACEI, angiotensin converting enzyme inhibitor; ARB, angiotensin receptor blocker; CI-AKI, contrast-induced acute kidney injury; P_1_, Amlodipine group vs Control group; P_2_, Amlodipine group vs Matched control group.

### Association Between Amlodipine and CI-AKI

The primary endpoint of CI-AKI occurred in 280 (10.50%) of the 2,667 group. The incidence of CI-AKI was significantly lower in amlodipine group (7.95%, 69 of 868, vs 11.74%, 211 of 1,798; *P *= 0.003). After matching, the same results were obtained between the amlodipine group and matched group (7.95%, 69 of 868, vs 11.87%, 103 of 868; *P *= 0.006). There was no significant difference between the two groups in the postoperative Scr levels between the two groups with or without matching. As is shown in the **Table 2**, amlodipine was associated with a significant shorten in hospital stay (7.08 ± 7.28 vs 7.77 ± 7.82; *P *= 0.027), and propensity score matching got the same result (7.08 ± 7.28 vs 7.81 ± 7.58; *P *= 0.040).

**Table 2:** The incidence of CI-AKI and the length of hospital stay in the amlodipine and control group.

Outcome	Amlodipine group (n=868)	Control group (n=1,798)	Matched control group (n=868)	*P_1_*	*P_2_*
CI-AKI	69 (7.95%)	211 (11.74%)	103 (11.87%)	0.003	0.006
Hospital stay	7.08 ± 7.28	7.77 ± 7.82	7.81 ± 7.58	0.027	0.040

CI-AKI, contrast-induced acute kidney injury; P_1_, Amlodipine group vs Control group; P_2_, Amlodipine group vs Matched control group.

**Figure 2:**
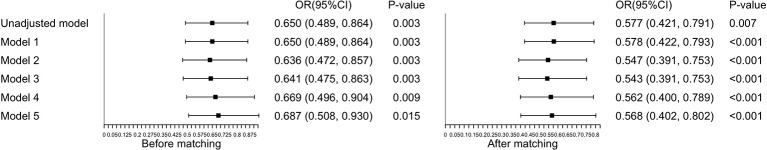
The association between amlodipine and CI-AKI in the adjusted model. Model 1 adjusted for age, sex and BMI. Model 2 adjusted for age, sex, BMI, baseline eGFR and Scr. Model 3 adjusted for age, sex, BMI, baseline eGFR, Scr, CKD and diabetes. Model 4 adjusted for age, sex, BMI, baseline eGFR, Scr, CKD, diabetes, Killip III, SBP, DBP, hyperlipidema and anemia. Model 5 adjusted for age, sex, BMI, baseline eGFR, Scr, CKD, diabetes, Killip III, SBP, DBP, hyperlipidema, anemia, asipilin, diuretic, ACEI, ARB, β blockers and alprostadil.

**Figure 3:**
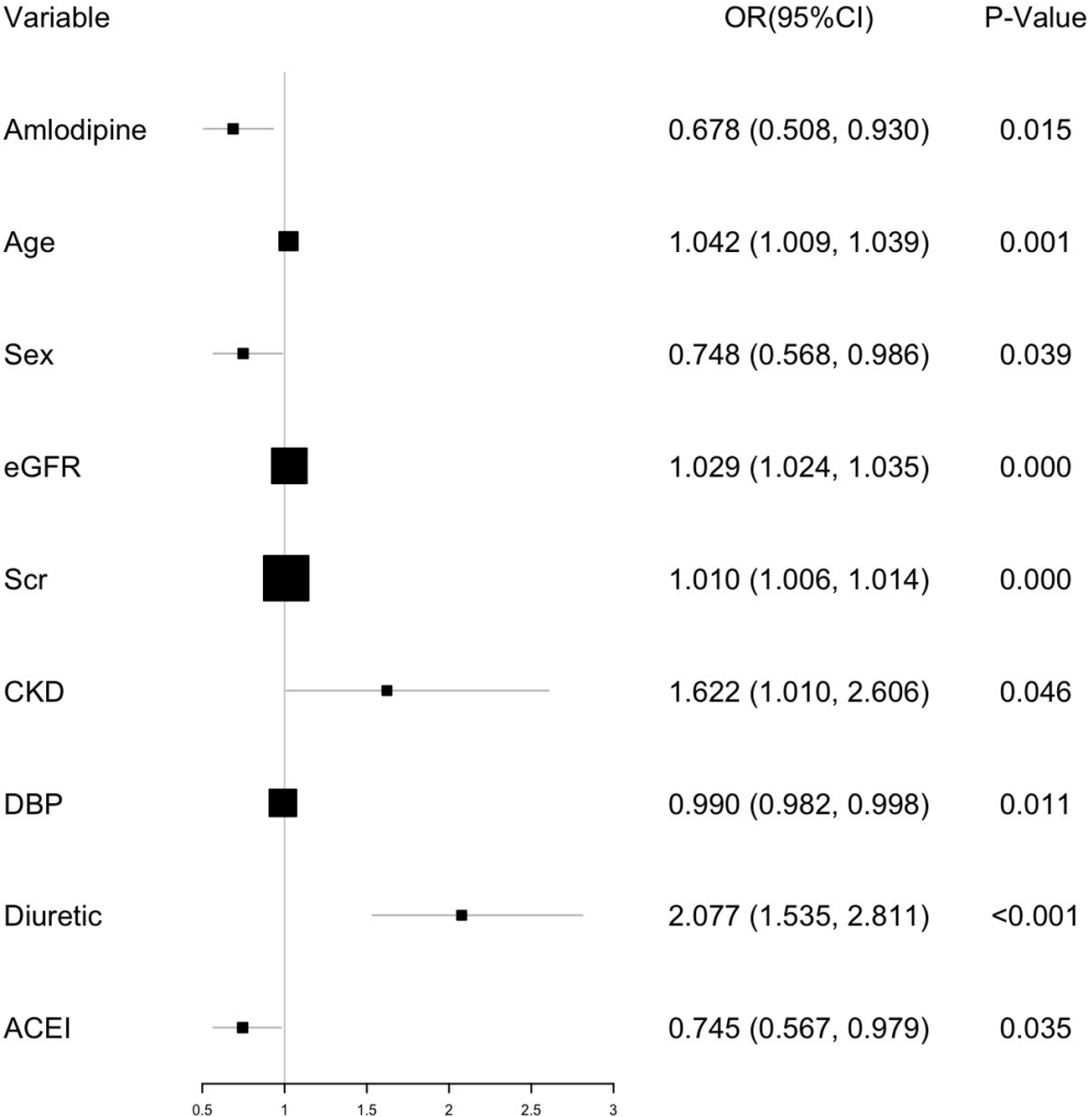
Multivariable analysis for predictors of CI-AKI before matching.

In the unadjusted model analysis, amlodipine reduced the risk of CI-AKI compared with controls [OR, 0.650; 95% confidence interval (CI), 0.489–0.864; *P *= 0.003], and this protection remained consistent after adjusting for baseline demographic characteristics, disease incidence, laboratory examination, and medication (OR, 0.687; 95% CI, 0.508–0.930; *P *= 0.015). After propensity score matching, amlodipine showed the same protection of CI-AKI whether using the unadjusted (OR, 0.577; 95% CI, 0.508–0.930; *P *= 0.007) or adjusted analysis (OR, 0.568; 95% CI, 0.402–0.802; *P *< 0.001, **Figure 2**). The multivariable logistic regression analysis also revealed that after amlodipine, age (OR, 1.204; *P* = 0.001), sex (OR, 0.748; *P* < 0.001), GFR (OR, 1.029; *P* < 0.001), Scr (OR, 1.010; *P *< 0.001), CKD (OR, 1.622; *P *= 0.046), DBP (OR, 0.990; *P* = 0.011), diuretic (OR, 2.077; *P *< 0.001), and ACEI (OR, 0.745; *P *= 0.035) were also independent predictors of CI-AKI development (**Figure 3**).

### Subgroup Analysis and Duration–Response Analysis

**Figure 4:**
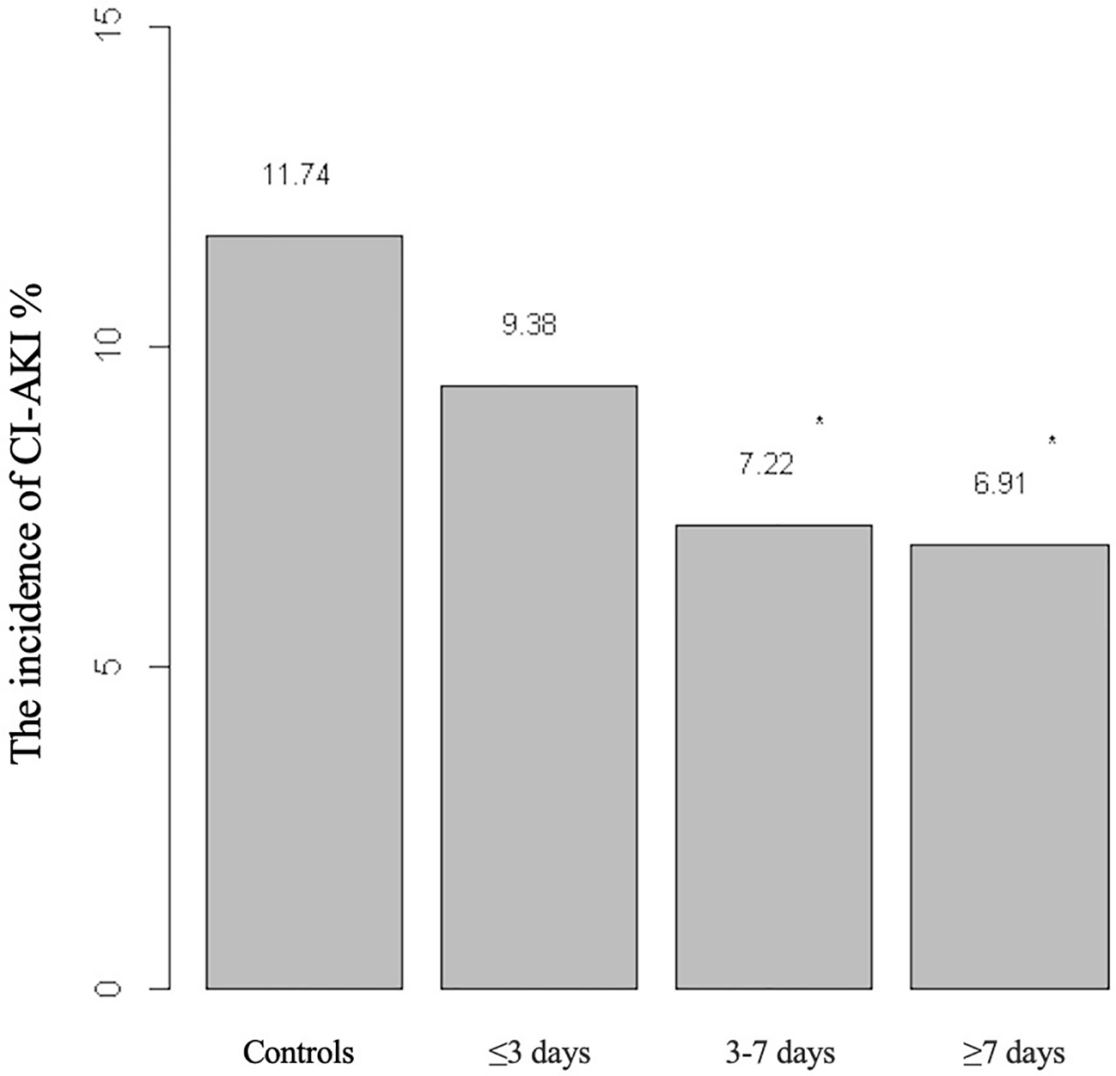
Risk of CI-AKI associated with increasing duration of amlodipine. *P < 0.05 vs. controls.

The efficacy of amlodipine on the prevention of incidence of CI-AKI in the subgroups was shown in **Table 3**. Before matching, a significant reduction in CI-AKI was observed in the elderly (OR, 0.571; 95% CI, 0.410–0.794; *P* < 0.001), diabetes groups (OR, 0.598; 95% CI, 0.368–0.969; *P* = 0.035), non-CKD group (OR, 0.654; 95% CI, 0.473–0.905, *P* = 0.01), and low-osmolar group (OR, 0.652; 95% CI 0.475–0.896, *P* = 0.008) but not CKD group (OR, 0.634; 95% CI, 0.349–1.152; *P* = 0.132) and iso-osmolar group (OR, 0.590; 95%, 0.282–1.234; *P* = 0.157). However, after matching, this result was found in the elderly (OR, 0.486; 95% CI, 0.338–0.699; *P* < 0.001) and those with diabetes (OR, 0.549; 95% CI, 0.324–0.930; *P* = 0.024), CKD (OR, 0.480; 95% CI, 0.253–0.911; *P*=0.023), non-CKD (OR, 0.612; 95% CI, 0.426–0.880, *P*=0.008), and low-osmolar (OR, 0.554; 95% CI 0.391–0.785, *P*=0.001), but not in iso-osmolar group (OR, 0.648; 95%, 0.281–1.491; *P*=0.305). We also investigated the duration–response relationship between duration of amlodipine therapy and risk of CI-AKI (**Figure 4**). It was found that as the duration of amlodipine increased from 3 to 7 days to greater than 7 days, there was a decrease risk of the CI-AKI incidence (11.74%, 9.38%, 7.22%, 6.91%).

**Table 3:** The association between amlodipine and CI-AKI in the subgroups.

Outcome	Amlodipine group	Control group	Matched control group	*P_1_*	*P_2_*
No. of Diabetes	296	500	285		
CI-AKI	25 (8.45%)	67 (13.40%)	41 (12.89%)		
OR		0.598 (0.368–0.969)	0.549 (0.324–0.930)	0.035	0.024
No. of CKD	205	382	200		
CI-AKI	16 (7.80%)	45 (11.78%)	30 (13.79%)		
OR		0.634 (0.349–1.152)	0.480 (0.253–0.911)	0.132	0.023
No. of non-CKD	663	1,416	668		
CI-AKI	53 (7.99%)	166 (11.72%)	83 (12.43%)		
OR		0.654 (0.473–0.905)	0.612 (0.426–0.880)	0.01	0.008
No. of elderly	665 (7.52%)	1,331 (12.47%)	635 (14.33%)		
CI-AKI	50	166	91		
OR		0.571 (0.410–0.794)	0.486 (0.338–0.699)	<0.001	<0.001
No. of low-osmolar	673	1,474	708		
CI-AKI	55 (8.17%)	177 (12.01%)	98 (13.84%)		
OR		0.652 (0.475–0.896)	0.554 (0.391–0.785)	0.008	0.001
No. of iso-osmolar	151	298	152		
CI-AKI	10 (6.62%)	32 (10.74%)	15 (9.87%)		
OR		0.590 (0.282–1.234)	0.648 (0.281–1.491)	0.157	0.305

CI-AKI, contrast-induced acute kidney injury; *P_1_*, Amlodipine group vs Control group; *P_2_*, Amlodipine group vs Matched control group.

### Survival Analysis

**Figure 5:**
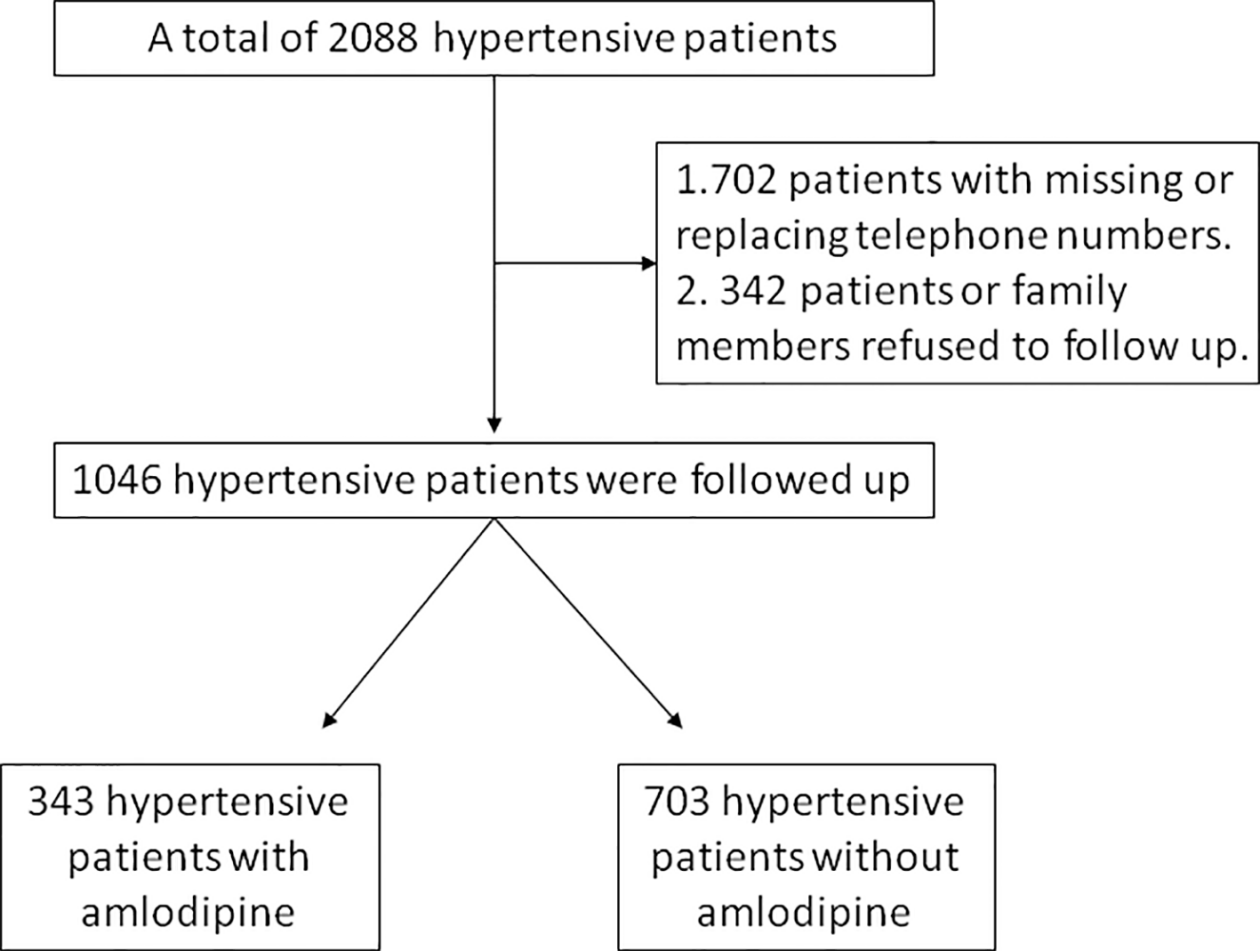
Flow diagram of survival analysis in this study.

**Figure 6:**
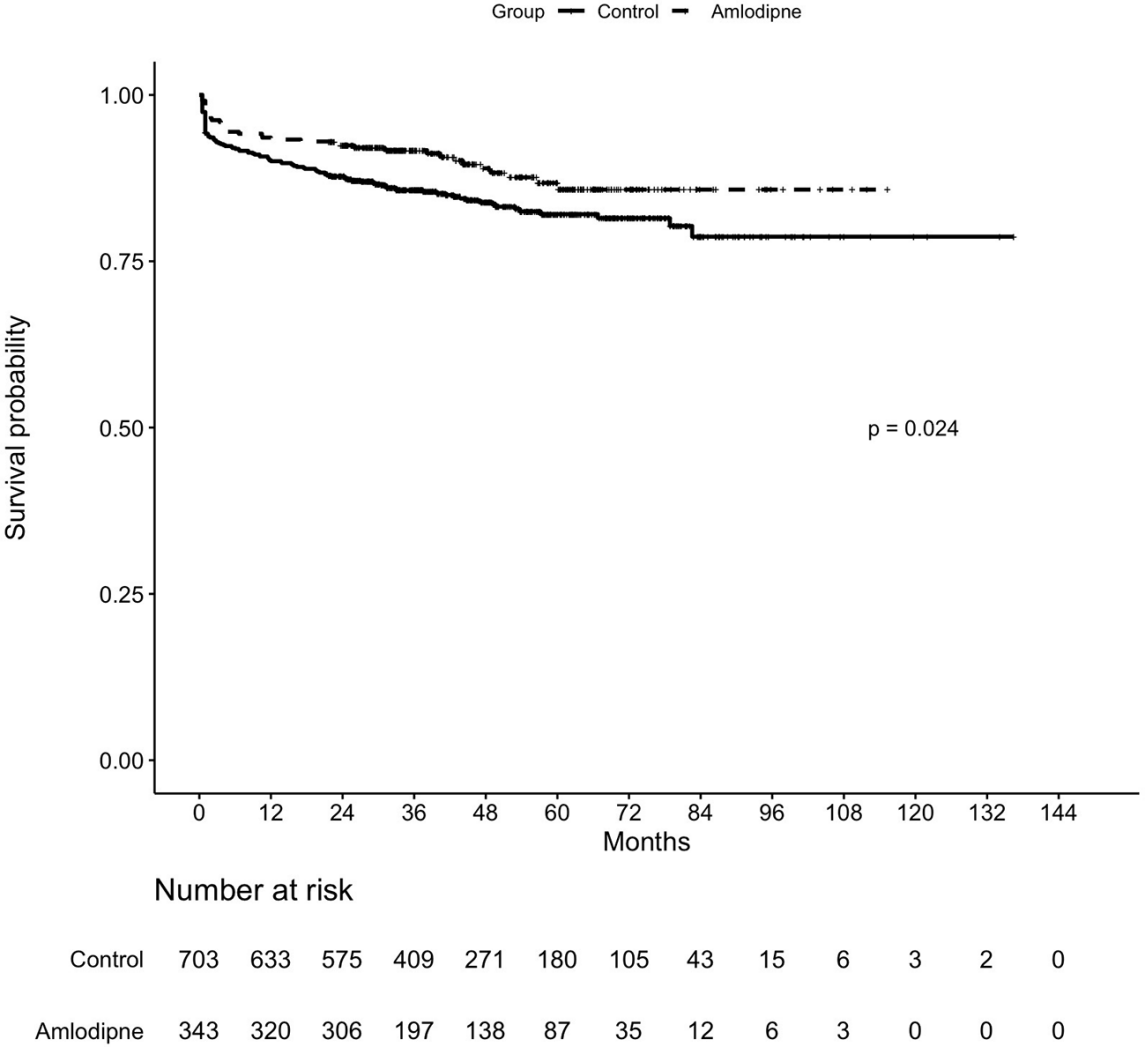
Kaplan–Meier estimates for overall survival in hypertensive patients with contrast administration receiving amlodipine vs controls.

A total of 1,046 patients were followed up for survival analysis including 343 amlodipine users and 703 non-users, 702 patients failed to follow up because missing or replacing telephone numbers, and 342 patients or family members refused (**Figure 5**). The data regarding overall survival were analyzed at a cutoff date of December 26, 2018, with a median follow-up of 40.85 months. The overall mortality was significantly lower among amlodipine users (10.79%) than controls (16.07%, *P*=0.028), the significant difference was found in survival between them (*P*=0.024, log-rank test, **Figure 6**). Upon univariate analysis, amlodipine use before CM was associated with longer overall survival (HR, 0.654; 95% CI, 0.451–0.948; *P*=0.025, **Table 4**). This trend persisted after adjusting for age, sex, BMI, baseline eGFR, baseline Scr, CKD, and diabetes (HR, 0.623; 95% CI, 0.430–0.908; *P*=0.014, **Table 4**).

**Table 4:** The association between amlodipine use and overall survival.

Variables	No amlodipine	Amlodipine	*P*
HR_unadjusted_ (95% CI)	1.00	0.654 (0.451–0.948)	0.025
HR_adjusted__1_ (95% CI)	1.00	0.657 (0.453–0.953)	0.027
HR_adjusted__2_(95% CI)	1.00	0.632 (0.435–0.918)	0.016
HR_adjusted__3_ (95% CI)	1.00	0.623 (0.430–0.908)	0.014

HR _adjusted__1_ adjusted for age, sex, and BMI; HR_adjusted__2_ adjusted for age, sex, BMI, baseline eGFR, and Scr; HR_adjusted__3_ adjusted for age, sex, BMI, baseline eGFR, baseline Scr, CKD, and diabetes.

## Discussion

For the first time, our present study directly addresses the prevention of amlodipine for CI-AKI in the hypertensive population which is a huge group with high risk of CI-AKI. The major finding of this study was that in patients with hypertension, amlodipine treatment prior to exposure to CM was able to reduce the risk of CI-AKI, before and after propensity matching and additional adjustment, and the same results were found in the diabetes, CKD, and the elderly subgroups. What's more, the treatment with amlodipine resulted in longer overall survival than controls. Moreover, the longer the use of amlodipine, the lower the incidence, but the difference was not significant.

Hypertensives is a huge group in high risk of CI-AKI. Ischemic and hypoxic of kidney has been reported as an important pathogenesis of CI-AKI, leading to inflammation, ischemia, and apoptosis. Hypertension remains a powerful prognostic marker for this condition. (Palit et al., 2015; James et al., 2015) Disorders of vasoactive substances, RAS activation, changes in inflammatory factors, and increased reactive oxygen species were involved in the mechanisms of renal damage caused by hypertension. (Pietri et al., 2015) Endothelial function and arterial stiffness changed with a decrease in nitric oxide (NO) in inflammation-induced hypertension as inflammation inducing available NO decrease in hypertension. (Boos and Lip, 2006) Oxidative stress also appears to be an important feature of NO reduction and is aggravated by an increase in circulating angiotensin II (Ang II) levels. (Hansell et al., 2013) Hypertension, combined with elevated levels of Ang II, and oxidative stress events, leading to renal damage.

Unfortunately, no specific therapeutic regimens exist to cure CI-AKI effectively. Thus, it is important to take precautions before CM administration. Amlodipine, one of the commonest CCB drugs for hypertension, has been reported to maintain renal function in patients with hypertension. (Nakamura et al., 2010; Honma et al., 2016) However, no study has reported the relationship between amlodipine and CI-AKI in hypertensives. The available data of these studies on the use of CCBs for the prevention of CI-AKI are contradictory and have not been clarified yet. Some studies have shown that CCB have a protective effect on the kidney and can prevent or reduce acute kidney injury caused by drugs *in vivo* and *vitro*. (Sepehr-Ara et al., 2010; Sepehr-Ara et al., 2011) A meta-analysis concluded that ACEI/ARB plus CCB (including amlodipine) is stronger than ACEI/ARB plus diuretics for maintaining renal function in hypertensive patients. (Cheng et al., 2016) The study by Russo et al. found that the renal plasma flow and GFR were increased in the group receiving nifedipine prior to injection of CM (n=30). (Russo et al., 1990) However, some other studies have reported the opposite. Oguzhan et al. objected to the benefit of a combination of an ARB and CCB in the treatment of CI-AKI in 90 CKD patients, the incidence of CI-AKI in patients undergoing coronary angiography received hydration along with amlodipine/valsartan (n = 45) increased when comparing to controls (n = 45), 17.8% vs 6.7%. (Oguzhan et al., 2013) In another study, among a total of 60 patients, isotonic sodium chloride, sodium bicarbonate, and isotonic sodium chloride with diltiazem application showed the same effect in prevention of CI-AKI. (Beyazal et al., 2014) But the sample size of the researches above is small, so in this study, we launched a much larger cohort: 2,667 hypertensive patients participated.

We first found amlodipine treatment before contrast exposure was able to reduce the risk of CI-AKI and prolong survival in the hypertensive population. Firstly, univariate analysis was carried out before matching. CKD and diabetes mellitus are known as the most important risk factors for CI-AKI, (Mccullough et al., 2016; Yin et al., 2017) and there were differences between the two groups at baseline eGFR, Scr, proportion of CKD, and diabetes, in order to reduce the effect of selection bias and potential confounding, we then matched controls on diabetes mellitus and renal function in 1:1 by propensity matching, in addition, age and sex were also matched. Because of the sample size, other antihypertensive drugs such as ACEI did not include in the matching analysis, we analyzed them in the conditional logistic regression. The protective effect of amlodipine was further confirmed by conditional logistic regression, adjusting for BMI, Killip III, SBP, DBP, complications, and combination medications. In this study, amlodipine was stable in reducing CI-AKI risk and shortening hospital stays, whether matched or adjusted. Finally, we conducted a subgroup analysis of the protective effects of amlodipine in high-risk groups such as diabetes, CKD, and the aged. Before matching, the morbidity was significant lower in the diabetes, non-CKD, low-osmolar, and aged amlodipine subgroups, and lower in subgroups of diabetes, CKD, non-CKD, low-osmolar, and the aged after matching.

The protective mechanism of amlodipine on CI-AKI is unclear, but some studies have studied the mechanism of renal protection by CCBs. It has been reported that renal tubular epithelial cells are associated with toxic effects of CM due to apoptosis, intrarenal hemodynamic disorder and medullary hypoxia, (Sadat, 2013) although the precise pathophysiological mechanism of CI-AKI also remains obscure. Current researches focus on contrast induced apoptosis caused by oxidative stress, increased Bcl-2/Bax, activation of caspase-3/9, and so on. (Romano et al., 2008; Gong et al., 2016; Kunak et al., 2016; Wang et al., 2017) Intracellular calcium overload which induced ROS overproduction and caspase-3 overexpression played an important role in the contrast-induced renal tubular cytotoxicity, and CCB plays a role in preventing calcium overload and inhibiting the opening of mitochondrial permeability transition pores (mPTP) by increasing calcium retention in protecting cardiomyocytes. (Ago et al., 2010; Mamou et al., 2015) Zhang et al. suggested that lacidipine treatment could protect human kidney cell against ischemia/reperfusion (I/R) injury by inhibiting the expression of Bax and Cytc proteins and by increasing Bcl-2 in an *in vitro* cell culture model to mimic I/R. (Zhang et al., 2013) And the caspase-3 pathway is involved in the protective mechanism. Caspase-3 activity peaked 30 min after ATP depletion and recovery, and it was attenuated by lacidipine. Yao et al. demonstrated that benidipine could ameliorate the AKI in rats due to the reduction of apoptosis in the tubular epithelial cells. (Yao et al., 2000).

What's more, substantial evidences showed that voltage-gated Ca^2+^ channel subtypes (L−, T−, N−, and P/Q−) exist in renal vessels and tubules, and blocking these channels play different effects on renal microcirculation. (Hayashi et al., 2007) L-Type calcium channel is widely distributed in renal vascular bed. L-Type CCB can significantly increase renal blood flow and glomerular filtration rate by blocking L-type calcium channel. Amlodipine, as a classical L-type calcium channel blocker, can also play a similar role. In summary, the underlying pathogenesis of amlodipine protection includes reduced apoptosis and inhibition of mPTP opening by preventing calcium overload, in addition, increase renal blood flow and glomerular filtration rate by blocking L-type calcium channel.

Cox proportional hazards models showed amlodipine before CM was a negative predictive risk factor for death after adjusting for age, sex, BMI, baseline eGFR, baseline Scr, CKD, and diabetes [HR 0.623, 95% CI (0.430–0.908), *P*=0.014]. CI-AKI has been shown associated with in-hospital need for dialysis (<1%), long-term kidney failure, and overall mortality (7–31%). (Azzalini et al., 2016) In this study, the mortality rate of hypertensive patients treated with contrast was 14.34%. A study involved 80 patients with CI-AKI after cardiac catheterization showed that CCB could reduce the mortality in CI-AKI patients. (Lai et al., 2013) Its role in ameliorating ischemic and toxic cell injury may play an important part in prolonging survival.

Multivariable analysis found that eGFR, Scr, and CKD were risk factors for CI-AKI. In previous studies, preexisting CKD has been reported to be the most important independent risk factor for CI-AKI. And the baseline eGFR, Scr, and potential kidney disease were included in many risk prediction models of CI-AKI. Our results were consistent with previous studies because baseline eGFR and Scr are two important indicators of renal function. Some studies have also shown that diabetes is a predictor of CI-AKI, and not all multivariable analyses have found it to be an independent risk factor because most diabetic patients suffered from CKD at the same time. In this study, diabetes was not a major risk factor for CI-AKI.

We also studied the effects of duration of amlodipine. When the duration is greater than 3 days, there was a lower risk of CI-AKI, although, this difference seems not significant. Thus, we advocate amlodipine at least three days in advance for a better preventive effect.

Some limitations of our analysis should be considered. First, this was a nonrandomized retrospective study, whose inherent weakness cannot be avoided despite robust propensity-score matching. Second, this study was conducted in a single center and may therefore be weaker in methodology than studies using multicenter sampled populations. Third, the effects of many drugs have not been ruled out, especially some renal protective drugs such as N-acetylcysteine. Fourth, in the survival analysis, some important information about patients were missing such as if some patients underwent further procedure with CM during the 144 months, AKI episodes, renal diseases, renal function, progression of hypertension, which result in the lax survival analysis. In the future prospective research, those information should be collected.

## Conclusions

Our data first demonstrate that the use of amlodipine may play a role in protecting patients from CI-AKI, and reducing the hospital stay in hypertensive population and the same results were found in the diabetes, CKD, and aged subgroups. We also found that amlodipine resulted in a statistically significant increase in overall survival among Chinese hypertensive patients with contrast for the first time. In addition, greater than 3 days duration and normal dose of levamlodipine 2.5 mg/qd or amlodipine 5.0 mg/qd before contrast administration show a better prevention from CI-AKI. This could provide guidance for medication for patients with hypertension.

## Data Availability Statement

The datasets generated for this study are available on request to the corresponding author.

## Ethics Statement

The studies involving human participants were reviewed and approved by the Medical Ethical Committee in the Third Xiangya Hospital of Central South University (No. 2016-S160). Written informed consent for participation was not required for this study in accordance with the national legislation and the institutional requirements.

## Author Contributions

W-JY and X-CZ conceived and designed the study. W-JY, X-CZ, and D-YL performed data acquisition and statistical analyses. W-JY and L-YZ managed the patient database. All authors were involved in the data interpretation and discussion of the results. W-JY, H-QY, and Y-JX prepared the figures. W-JY and X-CZ drafted the manuscript. All authors approved the final version of the manuscript.

## Funding

This study was supported by the National Natural Science Foundation of China (No. 81773822 and 81973400), the Hunan Traditional Chinese Medicine Research Program (No. 201760), the Hunan Pharmaceutical Society Scientific Research Funding Project (No. Hn2017003), and the Hunan Provincial Natural Science Foundation of China (No. 2018JJ6051).

## Conflict of Interest

The authors declare that the research was conducted in the absence of any commercial or financial relationships that could be construed as a potential conflict of interest.
